# Lactate transporter MCT4 regulates the hub genes for lipid metabolism and inflammation to attenuate intracellular lipid accumulation in non-alcoholic fatty liver disease

**DOI:** 10.1016/j.gendis.2025.101554

**Published:** 2025-02-15

**Authors:** Yannian Gou, Aohua Li, Xiangyu Dong, Ailing Hao, Jiajia Li, Han Xiang, Saidur Rahaman, Tong-Chuan He, Jiaming Fan

**Affiliations:** a Ministry of Education Key Laboratory of Diagnostic Medicine, and Department of Clinical Biochemistry, School of Clinical Laboratory Medicine, Chongqing Medical University, Chongqing 400016, China; b Western Institute of Digital-Intelligent Medicine, Chongqing 401329, China; c Molecular Oncology Laboratory, Department of Orthopaedic Surgery and Rehabilitation Medicine, The University of Chicago Medical Center, Chicago, IL 60637, USA

**Keywords:** Hepatic steatosis, Lactate, Lipid metabolism, Monocarboxylate transporter 4 (MCT4), Non-alcoholic fatty liver disease (NAFLD)

## Abstract

Non-alcoholic fatty liver disease (NAFLD) patients have multiple metabolic disturbances, with markedly elevated levels of lactate. Lactate accumulations play pleiotropic roles in disease progression through metabolic rearrangements and epigenetic modifications. Monocarboxylate transporter 4 (MCT4) is highly expressed in hepatocytes and responsible for transporting intracellular lactate out of the cell. To explore whether elevated MCT4 levels played any role in NAFLD development, we overexpressed and silenced MCT4 in hepatocytes and performed a comprehensive *in vitro* and *in vivo* analysis. Our results revealed that MCT4 overexpression down-regulated the genes for lipid synthesis while up-regulating the genes involved in lipid catabolism. Conversely, silencing MCT4 expression or inhibiting MCT4 expression led to the accumulation of intracellular lipid and glucose metabolites, resulting in hepatic steatosis. In a mouse model of NAFLD, we found that exogenous MCT4 overexpression significantly reduced lipid metabolism and alleviated hepatocellular steatosis. Mechanistically, MCT4 alleviated hepatic steatosis by regulating a group of hub genes such as *Arg2*, *Olr1*, *Cd74*, *Mmp8, Irf7*, *Spp1*, and *Apoe*, which in turn impacted multiple pathways involved in lipid metabolism and inflammatory response, such as PPAR, HIF-1, TNF, IL-17, PI3K-AKT, Wnt, and JAK-STAT. Collectively, our results strongly suggest that MCT4 may play an important role in regulating lipid metabolism and inflammation and thus serve as a potential therapeutic target for NAFLD.

## Introduction

Non-alcoholic fatty liver disease (NAFLD) is a series of chronic liver diseases that are mainly characterized by excessive lipid deposition in hepatocytes except alcohol and other well-defined liver-damaging factors. It can be divided into two stages: non-alcoholic fatty liver and non-alcoholic steatohepatitis, which can ultimately progress to cirrhosis and hepatocellular carcinoma.^1^ NAFLD is the second largest cause of end-stage liver disease and liver transplantation in Europe and the United States, with increasing worldwide incidence of overweight and obesity, showing a trend to affect younger populations, especially in China.^2^, ^3^, ^4^ NAFLD patients are often accompanied by changes in glucose and lipid metabolism, and these metabolic pathways in turn play an important role in compensating or exacerbating disease progression.^5^^,^^6^

Lactate is a glucose metabolism intermediate, and is considered the main carbon source of the tricarboxylic acid cycle.^7^^,^^8^ Previous studies indicated that lactate levels in the liver and plasma significantly increase with the disease process in NAFLD mice and non-alcoholic steatohepatitis patients.^9^, ^10^, ^11^ It seems that the accumulation of lactate is inevitable during the development of NAFLD. Moreover, elevated lactate levels can promote histone lactylation through metabolic rearrangements and epigenetic modifications, which then play pleiotropic roles in the progression of a variety of diseases, including hepatic fibrosis, dyslipidemia, and NAFLD.^8^^,^^12^^,^^13^ However, further studies are needed to investigate the role of lactate metabolic processes in NAFLD.

Monocarboxylate transporter 4 (MCT4, also known as SLC16A3) is a proton-dependent transporter that is expressed in all organs, and plays a key role in the transport of high levels of lactate across the plasma membrane, excreting endogenous lactates from the cell, effectively maintaining intracellular metabolism and pH homeostasis, and influencing carbohydrate and lipid metabolism.^14^^,^^15^ It is found that down-regulated MCT4 promoted diabetes-induced endothelial cell injury.^16^ Obesity was found to lead to impaired MCT4 expression in rat sarcomere vesicles and skeletal muscle, and mouse testes, while the expression of MCT4 in brains of obese mice was significantly increased.^17^, ^18^, ^19^, ^20^ Besides, our previous study found that syrosingopine, an MCT4 inhibitor, could significantly promote liver fibrosis.^21^ Consistently, MCT4 is highly expressed in patients with hepatocellular carcinoma and non-alcoholic steatohepatitis,^22^ suggesting that MCT4 may play an important role in regulating lipid metabolism and inflammatory response. Nevertheless, the exact role of MCT4 in NAFLD progression is unknown.

In this study, we investigated the role of MCT4 in NAFLD *in vitro* and *in vivo*. We analyzed publicly available transcriptomic datasets from patients with NAFLD and clinical samples and mouse models and found that MCT4 expression was associated with NAFLD progression. We demonstrated that increased MCT4 expression up-regulated numerous genes involved in lipid metabolism, reducing lipid accumulation; whereas silencing or inhibition of MCT4 induced the accumulations of lipid and glucose metabolites *in vitro*. The protective role of MCT4 in NAFLD was further confirmed by the finding that exogenous MCT4 reduced hepatic steatosis in a mouse high-fat model. Transcriptome sequencing analyses revealed that MCT4 alleviated NAFLD by regulating the hub genes involved in lipid metabolism and inflammatory response such as *Spp1*, *Apoe*, *Arg2*, *Olr1*, *Mmp8*, *Irf7*, and *Cd74*. Thus, our findings suggest that MCT4 may be explored as a potential therapeutic target for NAFLD treatment.

## Materials and methods

### Analysis of GEO datasets

Publicly available datasets GSE162694,^23^ GSE167523,^24^ and GSE174478^25^ were downloaded from Gene Expression Omnibus (GEO) (https://www.ncbi.nlm.nih.gov/geo/). Raw-count information was converted to transcript per million (TPM) using R (4.2.2). The results were visualized in GraphPad Prism 8. Spearman's correlation was performed between *MCT4* and other genes with embedded functions. When the correlation coefficient *r* > 0.3 and *P* < 0.05, it was considered correlated.

### The use of clinical samples

The hepatocellular carcinoma samples with NAFLD and adjacent non-tumor samples without NAFLD were purchased from the Department of Pathology, The First Affiliated Hospital of Chongqing Medical University.

### Cell culture and chemicals

The immortalized mouse E12.5 hepatic progenitor cells (iHPx), human HEK293-derived lines 293pTP and RAPA cells were described previously.^26^, ^27^, ^28^ All cells were cultured in high glucose complete Dulbecco's modified Eagle's medium (DMEM) supplemented with 10% fetal bovine serum (WISENT INC., Cat# 086-150, Uruguay), 100 units of penicillin, and 100 μg/mL of streptomycin at 37 °C in 5% CO_2_ as described.^29^, ^30^, ^31^ To induce hepatocyte steatosis, 0.05 mM oleic acids were used to supplement the medium for varied periods as reported.^30^^,^^32^ Syrosingopine (Cat# S9907, CAS 84-36-6) and BAY-8002 (Cat# S8747, CAS 724440-27-1) were purchased from Selleck Chemicals. VB124 (Cat# HY-139665, CAS 2230186-18-0) was purchased from MedChemExpress. Syrosingopine, BAY-8002, and VB124 were dissolved in dimethyl sulfoxide (DMSO) to prepare for a stock solution of 10 mM and was kept at −80 °C. Unless indicated otherwise, all other chemicals were purchased from Sigma–Aldrich (St Louis, MO), Thermo Fisher Scientific (Pittsburgh, PA), or Solarbio (Beijing, China).

### WST-1 cell proliferation assay

WST-1 assay was conducted as described.^33^ Briefly, subconfluent iHPx cells were seeded in 96-well plates (4000 cells/well) and treated with different concentrations of syrosingopine, BAY-8002, and VB124. At the indicated time points, the Premixed WST-1 Reagent (Takara Bio USA, Mountain View, CA) was added, and incubated at 37 °C for 120 min, followed by reading absorbance at 450 nm using a microplate reader (Biotek, EON, USA) as described.^34^^,^^35^ Each assay condition was performed in triplicate.

### RNA extraction and touchdown-quantitative real-time PCR (TqPCR)

Total RNA was isolated by using the TRIZOL Reagent (Invitrogen, China), and reverse-transcribed using hexamer and M-MuLV reverse transcriptase (New England Biolabs, Ipswich, MA). The cDNA products were subsequently used as TqPCR templates. Gene-specific PCR primers were designed using the Primer3 program (Table S1). TqPCR was carried out using 2x SYBR Green qPCR Master Mix (Bimake, Shanghai, China) on a CFX-Connect unit (Bio-Rad Laboratories, Hercules, CA) as described.^36^^,^^37^ All experiments were done in triplicate. *Gapdh* was used as a reference gene. Quantification of gene expression was carried out using the 2^−ΔΔCq^ method as described.^21^^,^^29^

### Oil red O staining

iHPx cells were seeded in 24-well culture plates and treated with different conditions for indicated time points. Alternatively, frozen sections from freshly prepared liver tissues in C57BL/6J mice were washed with phosphate buffer saline to remove the embedding agents. The oil red O staining was carried out as previously reported.^30^^,^^38^ Specifically, both cultured cells and frozen sections were fixed with 4% paraformaldehyde for 15 min, briefly incubated in 60% isopropanol, and then stained with freshly prepared oil red O solution for 5 min, followed by phosphate buffer saline washes. The staining results were recorded under a bright field microscope. Each assay condition was done in triplicate.

### Bodipy 493/503 staining

Bodipy 493/503 fluorescent detection of lipid droplets was carried out as reported.^30^^,^^38^^,^^39^ Cells were seeded in 24-well culture plates and with different conditions for indicated time points. The cells were fixed with 4% paraformaldehyde for 15 min, lipid droplets were stained with 30 μmol/L BODIPY493/503 (Sigma–Aldrich), and the nuclei were counter-stained with DAPI for 5 min, followed by phosphate buffer saline washes. The results were recorded under a fluorescence microscope. Each assay condition was done in triplicate.

### Construction and amplification of the recombinant adenoviruses Ad-MCT4, AdR-siMCT4, Ad-GFP, and Ad-RFP

All recombinant adenoviruses were constructed using the AdEasy technology as described.^40^, ^41^, ^42^ The Ad-MCT4 co-expresses human MCT4 and GFP, whereas AdR-siMCT4 co-expresses mouse siMCT4 and RFP. An analogous adenovirus expressing GFP or RFP only (Ad-GFP or Ad-RFP) was used as a mock virus control.

### Mouse model of NAFLD

The use and care of experimental animals were approved by the Research Ethics and Regulations Committee of Chongqing Medical University, Chongqing, China. All experimental procedures followed the approved guidelines. The mouse model of NAFLD was established as previously described.^30^^,^^45^^,^^46^ Briefly, 20 mice (C57BL/6J, male, 4 weeks old) were obtained from and housed in the Experimental Animal Research Center of Chongqing Medical University. The mice were randomly divided into two groups, the high-fat diet group was fed the 45% fat diet (MD12032, Medicience Ltd., China), whereas the control group was fed the 10% fat diet (MD12031, Medicience Ltd., China). Five mice from each group were sacrificed at week 16 and week 24, respectively.

For studying the effect of MCT4 on NAFLD, 10 mice (C57BL/6J, male, 4 weeks old) were fed with the 60% fat diet (XTHF60, Jiangsu Xietong Pharmaceutical Bio-engineering Co., Ltd., China). The mice were randomly divided into two groups, in which 5 mice were intrahepatically injected with 10^10^ pfu of Ad-GFP (in phosphate buffer saline, 30 μL in total per mouse) and the other with 10^10^ pfu of Ad-MCT4 (in phosphate buffer saline, 30 μL in total per mouse). The Ad injections were repeated once every 5 days. The dynamic body weight of mice was recorded every week. At 8 weeks, mice were sacrificed, and both serum and liver samples were retrieved for biochemical analysis and histologic evaluation, respectively.

### Determination of cellular, serum, and liver tissues levels of lactates, pyruvates, glucose, free fatty acids, and triglycerides

Cell preparation, mouse cardiac blood, and liver tissues collection were carried out as previously described.^21^^,^^43^^,^^44^ Briefly, prepared cells (5 × 10^6^ cells) were lysed with 500 μL of 1%–2% Triton X-100 in phosphate buffer saline for 30 min. The cardiac blood samples were centrifuged at 3500 revolutions per minute at room temperature for 10 min and the upper portion of the serum samples was collected. Alternatively, collected liver samples were homogenized in saline according to the manufacturer's protocol. All cell lysate, serum, and tissue samples were used for assessing the following commercial kits: pyruvate (Cat# A081-1-1, Nanjing Jiancheng Bioengineering Institute, China), glucose (Cat# A154-1-1, Nanjing Jiancheng Bioengineering Institute), lactate (Cat# A019-2-1, Nanjing Jiancheng Bioengineering Institute), and triglyceride (Cat# A110-1-1, Nanjing Jiancheng Bioengineering Institute), and free fatty acid (Cat# A042-1-1, Nanjing Jiancheng Bioengineering Institute).

### Hematoxylin & eosin staining and immunohistochemical staining

The retrieved liver samples were fixed and subjected to paraffin embedding, followed by sectioning. The sections were deparaffinized and subjected to hematoxylin & eosin staining (Solarbio, Cat# G1120) and immunohistochemical staining as described.^44^^,^^47^ For immunohistochemical staining, the tissue sections were deparaffinized, rehydrated, antigen-retrieval treated, blocked, and incubated overnight with primary antibodies against ARG2 (1:50–1:500 dilution; Santa; Cat# sc-393496) and MCT4 (1:1000–1:4000 dilution; Proteintech; Cat# 22787-1-AP), followed by staining with biotin-labeled goat anti-rabbit IgG or anti-mouse IgG/streptavidin-HRP kit (SP Kit, PV-9000, ZSGB-Bio, China). Minus primary antibody and/or rabbit IgG and mouse IgG were used as negative controls. The staining results were recorded under a bright field microscope (Leica, DM4B).

### Next-generation RNA-sequencing analysis

The mRNA-sequencing analysis was conducted as previously described.^38^ Briefly, exponentially growing iHPx cells were oleic acid-induced and simultaneously infected with Ad-MCT4, AdR-siMCT4, or Ad-RFP for 48 h. Total RNA was isolated with the TRIZOL Reagent (Invitrogen, China) and subjected to mRNA sequencing (Knorigene Technologies, Chongqing, China). The sample matrix was collated using R (4.2.2). Genes with fold change > 1.2 or < 0.8 and *P*-value < 0.05 were assigned as differentially expressed genes (DEGs). Venn diagrams and the clustering heatmap were drawn as previously described.^38^ Gene Ontology (GO) and Kyoto Encyclopedia of Genes and Genomes (KEGG) pathway enrichment analyses were also performed as previously described.^38^ Gene set enrichment analysis (GSEA) was generated at https://www.bioinformatics.com.cn/, an online platform for data analysis. The ggplot2 package was used to visualize the results in R. The GSEA terms with *P* value < 0.05 and *q* value < 0.25 were considered significantly enriched. Overlapping genes were analyzed using STRING (https://cn.string-db.org/) to obtain a protein–protein interaction relationship network. The relationship network was imported into Cytoscape (version v3.10.2) for visualization, the top 10 hub genes were identified using the “clustering coefficient” algorithm of the cytoHubba plugin, and the highest-scoring gene module was selected for subsequent analysis.

### Western blotting analysis

Western blotting was carried out as previously described.^21^^,^^31^^,^^48^ Briefly, iHPx cells treated with various conditions and lysed in RIRA lysis buffer containing phosphatase inhibitors and protease inhibitors. The proteins were separated by 10% SDS-PAGE and transferred to PVDF membranes, followed by being blocked and incubated overnight with the primary antibodies against β-ACTIN (1:5000–1:50,000 dilution; Proteintech; Cat# 66009-1-Ig), ARG2 (1:100–1:1000 dilution; Santa; Cat# sc-393496), and MCT4 (1:2000–1:20,000 dilution; Proteintech; Cat# 22787-1-AP). Subsequently, the membranes were incubated with secondary antibodies (1:5000 dilution; ZSGB-BIG; peroxidase-conjugated rabbit anti-goat IgG or peroxidase-conjugated goat anti-mouse IgG, Cat# ZB-2301 or 2305) conjugated with horse radish peroxidase. Finally, immune-reactive signals were visualized with the enhanced chemiluminescence kit (Millipore, USA) and recorded using the Bio-Rad ChemiDoc Imager (Hercules, CA).

### Statistical analysis

Data were analyzed using GraphPad Prism 8 and presented as mean ± standard deviations. Statistical significance was determined by one-way analysis of variance and the student's *t*-test for the comparisons between groups. For correlation analysis, Spearman's correlation coefficient was used. *P* values < 0.05 were considered statistically significant.

## Results

### High MCT4 expression in hepatocytes is positively correlated with NAFLD

**Figure 1:**
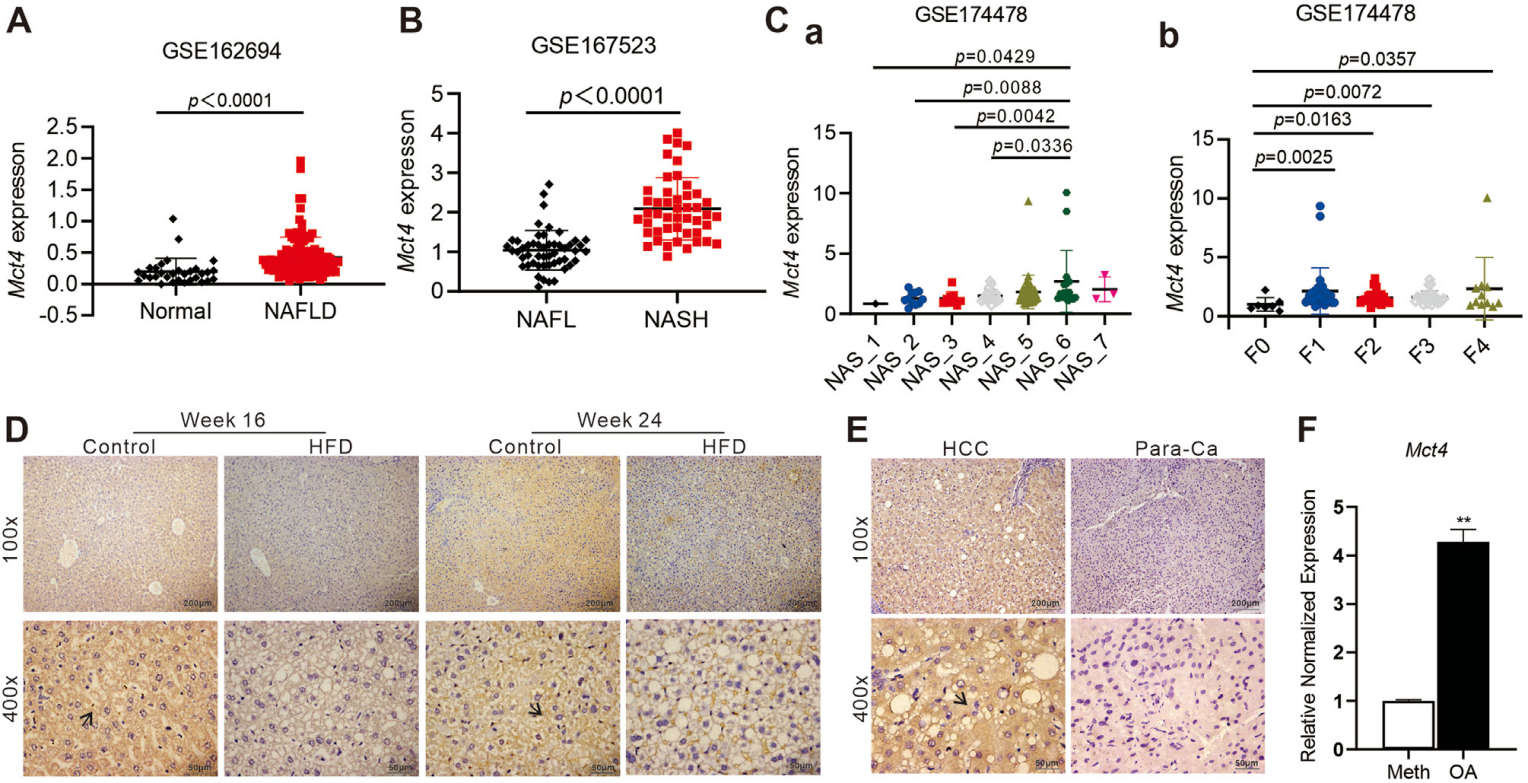
Correlation analysis between the expression of MCT4 in hepatocytes and NAFLD. **(A, B)** The scatter plots for *Mct4* TPM of Normal (*n* = 31) and NAFLD (*n* = 112) in the GSE162694 dataset (A), and of NAFL (*n* = 51) and NASH (*n* = 47) in the GSE167523 dataset (B). Unpaired, two-sided Mann–Whitney U test *P*-values are depicted in the plots, and the significant *P*-value cutoff was set at 0.05. The plots show the medians (black line), standard deviation, and *P*-values. **(C)** The scatter plots for *Mct4* TPM in GSE174478 (*n* = 94) in which patients were stratified by NAFLD activity score (NAS, left) or fibrosis score (F, right). *P*-values were obtained via a nonparametric two-stage Benjamini, Krieger, & Yekutieli false discovery rate (FDR) procedure. The mean expression (black line), standard deviation, and *P*-values are shown. **(D)** The immunohistochemical staining of protein levels of MCT4 in the livers of C57BL/6J mice fed with chow diet or high-fat diet (HFD). Representative positive stains are indicated with black arrows (100 × and 400 × ). **(E)** The immunohistochemical staining of MCT4 expression in hepatocellular carcinoma patients with NAFLD (HCC group) and adjacent non-tumor tissues without NAFLD (Para-Ca group). Representative positive stains are indicated with black arrows (100 × and 400 × ). Each assay condition was done in triplicate, and representative images were shown. **(F)** The expression of *Mct4* in oleic acid (OA)-induced lipid accumulation in hepatocytes. Subconfluent iHPx cells were stimulated with 0.05 mM OA (methanol as a vehicle control), total RNA was isolated at 24 h and subjected to touchdown-quantitative PCR analysis of *Mct4* expression. ∗∗*P* < 0.01, OA versus Methanol. MCT4, monocarboxylate transporter 4; NAFLD, non-alcoholic fatty liver disease; TPM, transcript per million; NAFL, non-alcoholic fatty liver; NASH, non-alcoholic steatohepatitis.

To investigate the correlation between MCT4 and NAFLD, we first analyzed hepatic gene expression of NAFLD patients in the GEO public datasets and revealed that *MCT4* (*SLC16A3*) mRNA expression was highly expressed in NAFLD patients (GSE162694) (Fig. 1A). In addition, the *MCT4* mRNA expression was significantly elevated when patients progressed from non-alcoholic fatty liver to non-alcoholic steatohepatitis (GSE167523) (Fig. 1B). Similarly, the analysis of transcriptomic dataset GSE174478 showed that higher *MCT4* mRNA expression was significantly associated with increased NAFLD activity score and fibrosis score (Fig. 1C). Interestingly, the histopathologic staining showed that NAFLD progression was aggravated in mice from 16 to 24 weeks, while hepatic MCT4 protein levels were significantly lower in the livers from mice fed with a high-fat diet (Fig. 1D; Fig. S1A). However, the hepatic MCT4 protein levels were significantly higher in liver tissues of patients with hepatocellular carcinoma and NAFLD than in the adjacent non-tumor tissues (Fig. 1E), suggesting that MCT4 expression levels may vary at different species and developmental stages of NAFLD. The negative control is shown in Figure S1B. Next, oleic acid was used to mimic hepatic steatosis in NAFLD *in vitro*.^30^^,^^49^ The *MCT4* mRNA expression in hepatocytes was significantly elevated after 24 h of oleic acid treatment (Fig. 1F). These results indicate that up-regulation of MCT4 is associated with the progression of NAFLD although the role of MCT4 expression during NAFLD development requires further investigation.

### Inhibition of MCT4 expression leads to more lipid accumulation in hepatocytes

**Figure 2:**
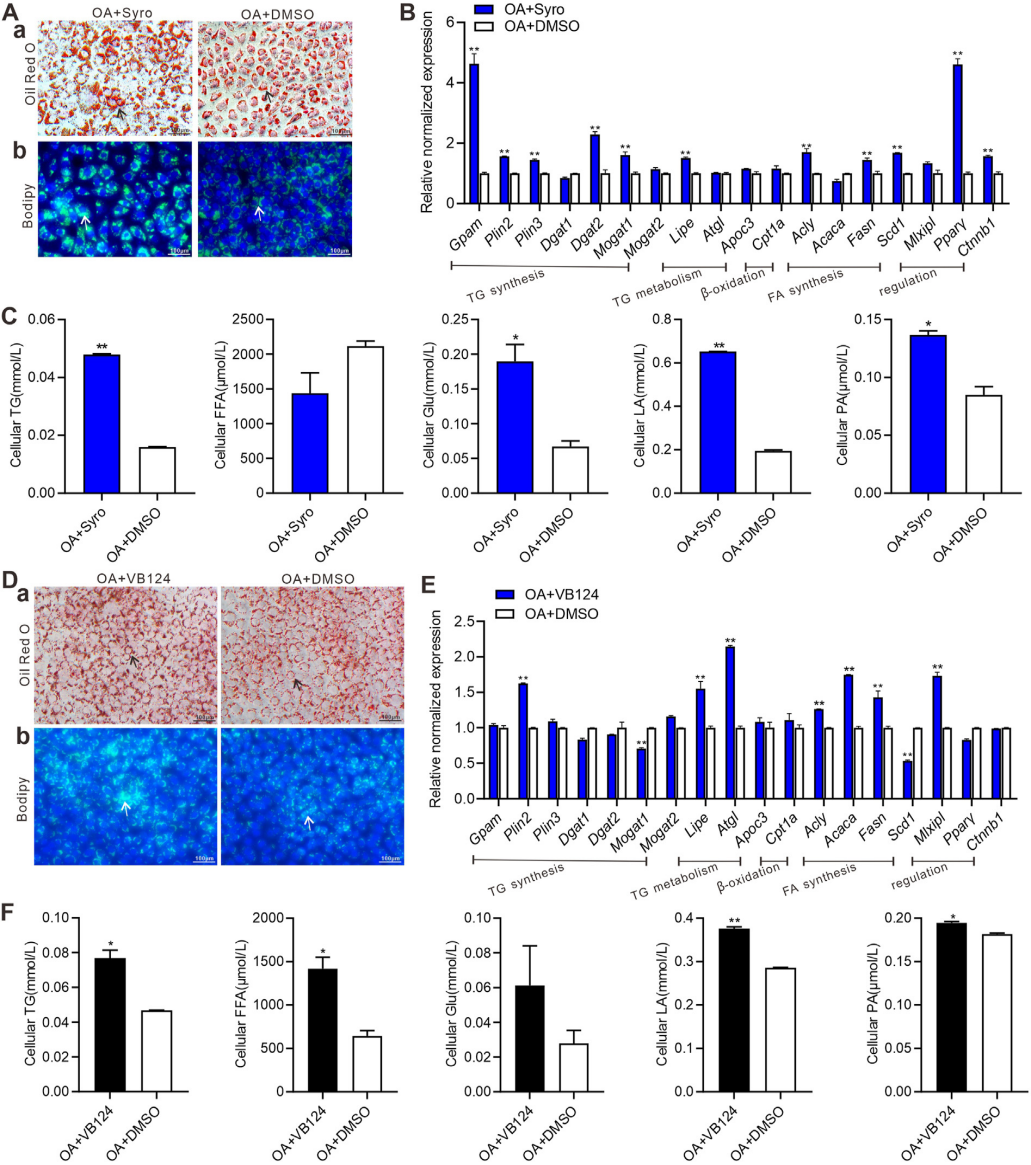
Syrosingopine and VB124 induce lipid accumulation in hepatocytes. Subconfluent iHPx were treated with 10 μM syrosingopine (abbreviated as Syro, DMSO as the solvent control) or DMSO respectively. Simultaneously, cells were stimulated with 0.05 mM oleic acid (OA) for the indicated time points. **(A)** The oil red O staining (a) and bodipy 493/503 staining (b) were done on day 5. Representative lipid droplets were indicated with arrows (200 × ). **(B)** Touchdown-quantitative PCR analysis of the expression of genes involved in lipolysis and lipogenesis in hepatocytes at 36 h. Expression of each target gene was calculated as a relative expression to *Gapdh*. ∗∗*P* < 0.01, OA + Syro versus OA + DMSO. **(C)** The cellular triglyceride (TG), free fatty acid (FFA), glucose (Glu), lactate (LA), and pyruvate (PA) levels were measured at day 5. ∗∗*P* < 0.01, ∗*P* < 0.05, OA + Syro versus OA + DMSO. Alternatively, subconfluent iHPx were treated with 10 μM VB124 or DMSO (DMSO as the solvent control), and 0.05 mM OA was also used to treat iHPx at the indicated time points. **(D)** The oil red O staining (a) and bodipy 493/503 staining (b) were done on day 5. Representative lipid droplets were indicated with arrows (200 × ). **(E)** Touchdown-quantitative PCR analysis of the expression of the genes involved in lipolysis and lipogenesis in hepatocytes at 36 h. Expression of each target gene was calculated as a relative expression to *Gapdh*. ∗∗*P* < 0.01, OA + VB124 versus OA + DMSO. **(F)** The cellular TG, FFA, Glu, LA, and PA levels were measured on day 5. ∗∗*P* < 0.01, ∗*P* < 0.05, OA + VB124 versus OA + DMSO. Each assay condition was done in triplicate, and representative images were shown.

Syrosingopine is used to inhibit both MCT4 and MCT1, but inhibits MCT4 more efficiently than MCT1.^21^^,^^50^ The optimal concentration of syrosingopine was screened by WST-1 and 10 μM was selected for further studies (Fig. S1C). The TqPCR and western blotting analyses showed that syrosingopine significantly inhibited MCT4 mRNA and protein expression (Fig. S1D, E). The oil red O staining and bodipy 493/503 staining showed that syrosingopine markedly increased lipid accumulation in hepatocytes at day 5 (Fig. 2A). The TqPCR analysis showed that syrosingopine could increase the expression of genes involved in the synthesis and regulation of triglycerides and fatty acids, including *Acly*, *Fasn*, *Scd1*, *Pparγ, Gpam*, *Plin2*, *Plin3*, *Dgat2*, *Mogat1*, and *Ctnnb1* at 36 h, and meanwhile, up-regulate the genes involved in lipid catabolism, such as *Lipe* in hepatocytes (Fig. 2B). Furthermore, we found that the intracellular levels of triglycerides, glucose, lactates, and pyruvates were significantly elevated after treated by syrosingopine than those in the control group at day 5 in hepatocytes (Fig. 2C).

VB124 is considered an MCT4-specific inhibitor and was used to inhibit the expression of MCT4.^51^ Similarly, the optimal concentration of VB124 was screened by WST-1, and 10 μM was selected for further studies (Fig. S1C). We similarly demonstrated that VB124 effectively suppressed MCT4 expression at the gene and protein levels (Fig. S1D, E). The oil red O staining and bodipy 493/503 staining showed that VB124 significantly increased lipid accumulation in hepatocytes at day 5 (Fig. 2D). The TqPCR analysis further showed that VB124 increased the expression of genes involved in triglyceride and fatty acid synthesis and regulation, including *Mlxipl*, *Acly*, *Acaca*, *Fasn*, and *Plin2*, and genes involved in lipid catabolism such as *Lipe* and *Atgl* at 36 h in hepatocytes (Fig. 2E). Meanwhile, intracellular triglyceride, free fatty acid, lactate, and pyruvate levels were significantly higher in the VB124 treated group than those in the control group at day 5 in hepatocytes (Fig. 2F).

We also used BAY-8002, a specific MCT1 inhibitor,^52^ to inhibit MCT1 expression in hepatocytes (Fig. S2A). The oil red O staining and bodipy 493/503 staining showed that BAY-8002 decreased lipid accumulation in hepatocytes at day 5 (Fig. S2B). The TqPCR analysis showed that BAY-8002 reduced the expression of genes involved in the synthesis and regulation of triglycerides and fatty acids, including *Acaca*, *Fasn*, *Scd1*, *Pparγ*, *Gpam*, *Dgat2*, *and Plin2*, while up-regulated the expression of *Dgat1*, *Mogat1*, *and Ctnnb1*, and down-regulated the genes involved in lipolysis, such as *Lipe*, *Atgl*, and *Cpt1a* at 36 h in hepatocytes (Fig. S2C). As expected, the intracellular levels of triglycerides, free fatty acids, glucose, and lactates were dramatically lower in the BAY-8002 treated group than those in the control group at day 5 in hepatocytes (Fig. S2D). These results indicate that MCT4 inhibition can up-regulate the expression of genes involved in triglyceride and fatty acid synthesis resulting in increased intracellular lipid accumulation.

### Silencing MCT4 leads to increased lipid accumulation while overexpression of MCT4 reduces lipid accumulation in hepatocytes effectively

**Figure 3:**
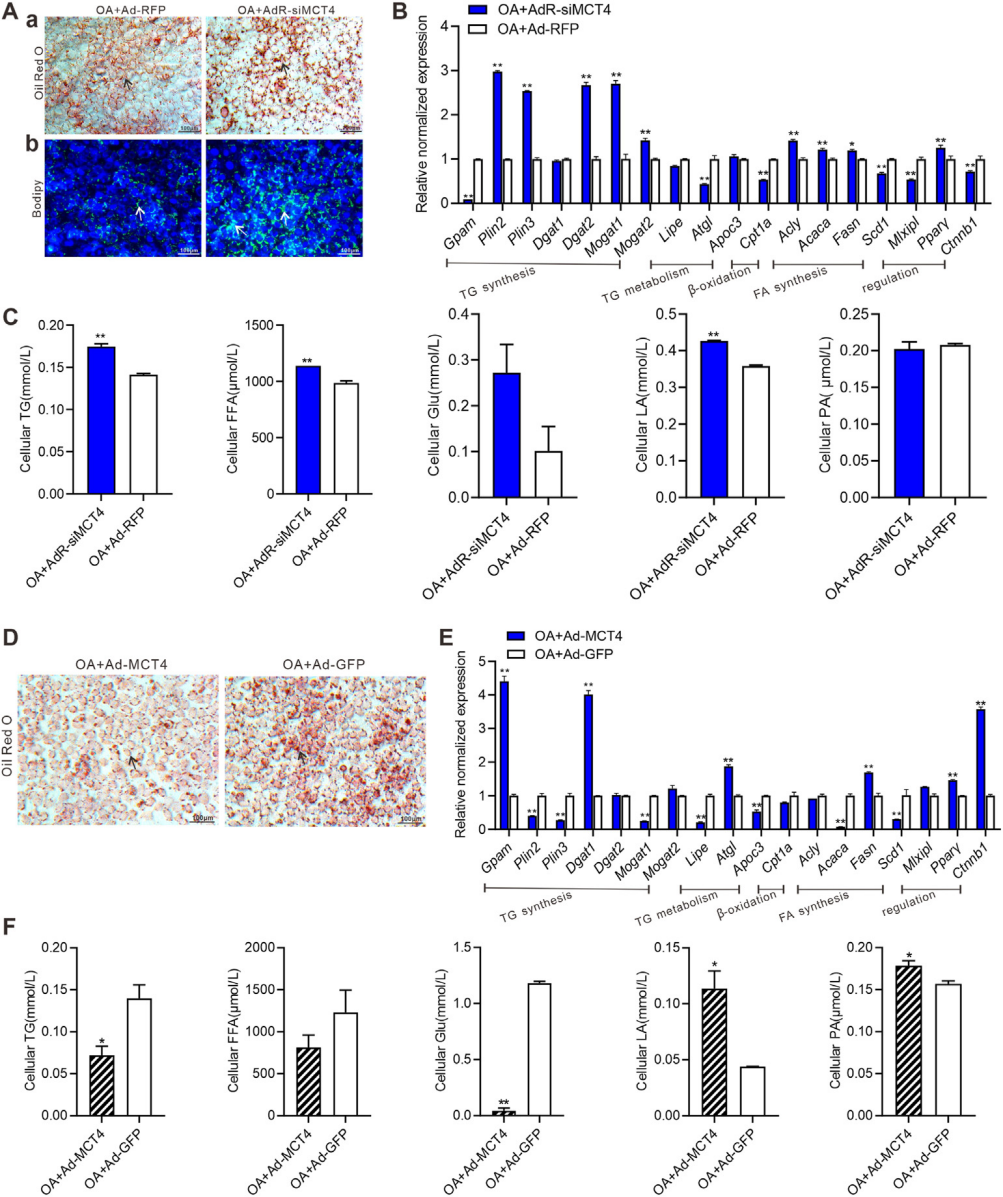
Silencing MCT4 induces lipid accumulation while exogenous MCT4 leads to decreased lipid accumulation in hepatocytes. Subconfluent iHPx were infected with AdR-siMCT4 or Ad-RFP and stimulated with 0.05 mM oleic acid (OA) for the indicated time points. **(A)** The oil red O staining (a) and bodipy 493/503 staining (b) were done on day 5. Representative lipid droplets were indicated with arrows (200 × ). **(B)** Touchdown-quantitative PCR analysis of the expression of the genes involved in lipolysis and lipogenesis in hepatocytes at 48 h. Expression of each target gene was calculated as a relative expression to *Gapdh*. ∗*P* < 0.05; ∗∗*P* < 0.01, OA + AdR-siMCT4 versus OA + Ad-RFP. **(C)** The cellular triglyceride (TG), free fatty acid (FFA), glucose (Glu), lactate (LA), and pyruvate (PA) levels were measured at day 5. ∗∗*P* < 0.01, OA + AdR-siMCT4 versus OA + Ad-RFP. Alternatively, subconfluent iHPx were infected with Ad-MCT4 or Ad-GFP and stimulated with 0.05 mM OA for the indicated time points. **(D)** The oil red O staining was done on day 7. Representative lipid droplets were indicated with arrows (200 × ). **(E)** Touchdown-quantitative PCR analysis of the expression of the genes involved in lipolysis and lipogenesis, and transcriptional regulation in hepatocytes at 48 h. Expression of each target gene was calculated as a relative expression to *Gapdh*. ∗∗*P* < 0.01, OA + Ad-MCT4 versus OA + Ad-GFP. **(F)** The cellular TG, FFA, Glu, LA, and PA levels were measured on day 5. ∗∗*P* < 0.01, ∗*P* < 0.05, OA + Ad-MCT4 versus OA + Ad-GFP. Each assay condition was done in triplicate, and representative images were shown. MCT4, monocarboxylate transporter 4.

We constructed the recombinant adenovirus AdR-siMCT4 to specifically silence the expression of mouse MCT4. The TqPCR and western blotting analyses verified the down-regulation of MCT4 expression in hepatocytes after AdR-siMCT4 treatment (Fig. S3A, panels a–c). The oil red O staining and bodipy 493/503 staining showed that silencing MCT4 significantly increased lipid accumulation at day 5 in hepatocytes (Fig. 3A). Furthermore, the TqPCR analysis revealed that siMCT4 led to the up-regulation of genes related to triglyceride and fatty acid synthesis, including *Acly*, *Acaca*, *Fasn*, *Pparγ*, *Plin2*, *Plin3*, *Dgat2*, *Mogat1*, and *Mogat2*, and the down-regulation of genes related to triglyceride and fatty acid catabolism, including *Atgl* and *Cpt1a* in hepatocytes at 48 h (Fig. 3B). The intracellular metabolites analysis showed that intracellular triglyceride, free fatty acid, and lactate levels were significantly higher in the AdR-siMCT4 group than in the control group in hepatocytes at day 5 (Fig. 3C).

Next, we constructed the recombinant adenovirus Ad-MCT4 to over-express MCT4, and verified successful overexpression at gene and protein levels (Fig. S3B, panels a–c). The oil red O staining showed that overexpression of MCT4 significantly decreased lipid accumulation in hepatocytes compared with the Ad-GFP treated group at day 7 (Fig. 3D). The TqPCR analysis revealed that overexpression of MCT4 down-regulated *Acaca*, *Scd1*, *Plin2*, *Plin3*, *Mogat1*, *Lipe*, and *Apoc3*, and up-regulated *Atgl*, *Fasn*, *Pparγ*, *Gpam*, *Dgat1*, and *Ctnnb1* (Fig. 3E). The intracellular triglyceride and glucose levels were also down-regulated while lactate and pyruvate levels were increased at day 5 (Fig. 3F). Taken together, these results suggest that MCT4 may play a protective role in the progression of hepatic steatosis.

### MCT4 inhibits NAFLD progression *in vivo*

**Figure 4:**
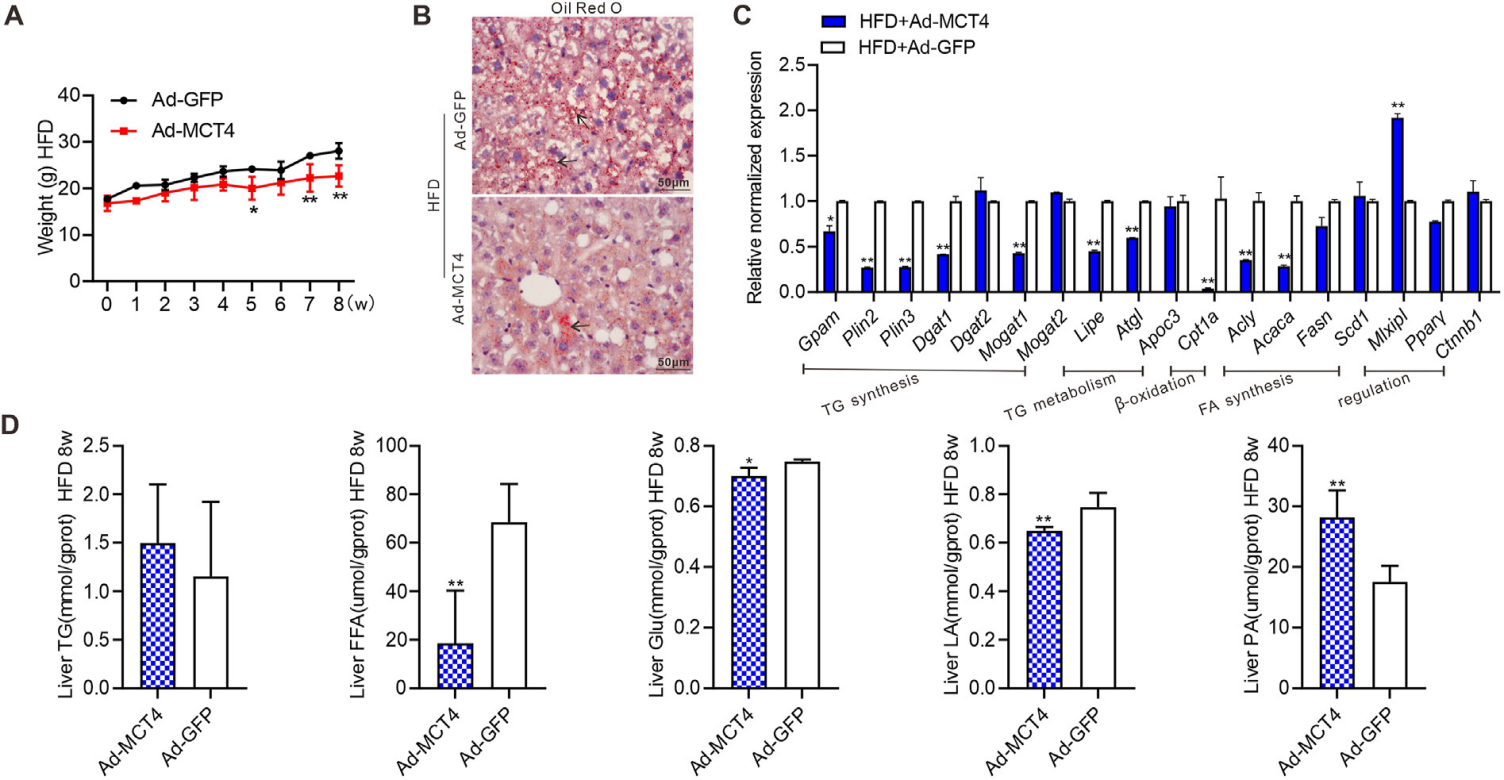
MCT4 inhibits the progress of NAFLD *in vivo*. A NAFLD model was built as described. Ad-MCT4 or Ad-GFP was intrahepatically injected once every 5 days. The mice were sacrificed at week 8. **(A)** The dynamic weight changes of mice. ∗∗*P* < 0.01, ∗*P* < 0.05, HFD + Ad-MCT4 versus HFD + Ad-GFP. **(B)** The retrieved liver tissues were subjected to oil red O staining. Representative positive stains are indicated with black arrows (400 × ). **(C)** Total RNA was isolated from the liver tissues of the mice and touchdown-quantitative PCR analysis of the expression of the genes involved in lipolysis and lipogenesis, and transcriptional regulation. The expression of each target gene was calculated as a relative expression to *Gapdh*. ∗∗*P* < 0.01, ∗*P* < 0.05, HFD + Ad-MCT4 versus HFD + Ad-GFP. **(D)** The cellular triglyceride (TG), free fatty acid (FFA), glucose (Glu), lactate (LA), and pyruvate (PA) levels were measured. ∗∗*P* < 0.01, ∗*P* < 0.05, HFD + Ad-MCT4 versus HFD + Ad-GFP. Each assay condition was done in triplicate, and representative images were shown. MCT4, monocarboxylate transporter 4; NAFLD, non-alcoholic fatty liver disease; HFD, high-fat diet.

We established a NAFLD mouse model as described,^30^^,^^32^ and Ad-MCT4 or Ad-GFP was intrahepatically injected every five days to investigate whether MCT4 would prevent and/or alleviate hepatic steatosis. We found that exogenous MCT4 markedly reduced mouse body weight since week 5 (Fig. 4A). Hematoxylin & eosin staining of the liver tissues revealed hepatocyte swelling and hepatic steatosis in the Ad-GFP treated group, and to a lesser extent in the Ad-MCT4 treated group (Fig. S3C). The oil red O staining showed lower lipid accumulation in Ad-MCT4 treated group compared with the Ad-GFP treated group (Fig. 4B). Exogenous expression of MCT4 down-regulated numerous lipolysis and lipogenesis genes related to triglycerides and fatty acids, including *Acly*, *Acaca*, *Gpam*, *Plin2*, *Plin3*, *Dgat1*, *Mogat1*, *Lipe*, *Atgl*, and *Cpt1a* (Fig. 4C). Although no significant changes in serum metabolite concentrations were observed (Fig. S3D), intrahepatic free fatty acid, glucose, and lactate levels were down-regulated and pyruvate level was increased (Fig. 4D). Collectively, these results strongly suggest that MCT4 overexpression can inhibit hepatic steatosis by down-regulating a range of hepatic lipid metabolism-related genes.

### MCT4 regulates hepatic lipid metabolism by driving multiple genes interactions

**Figure 5:**
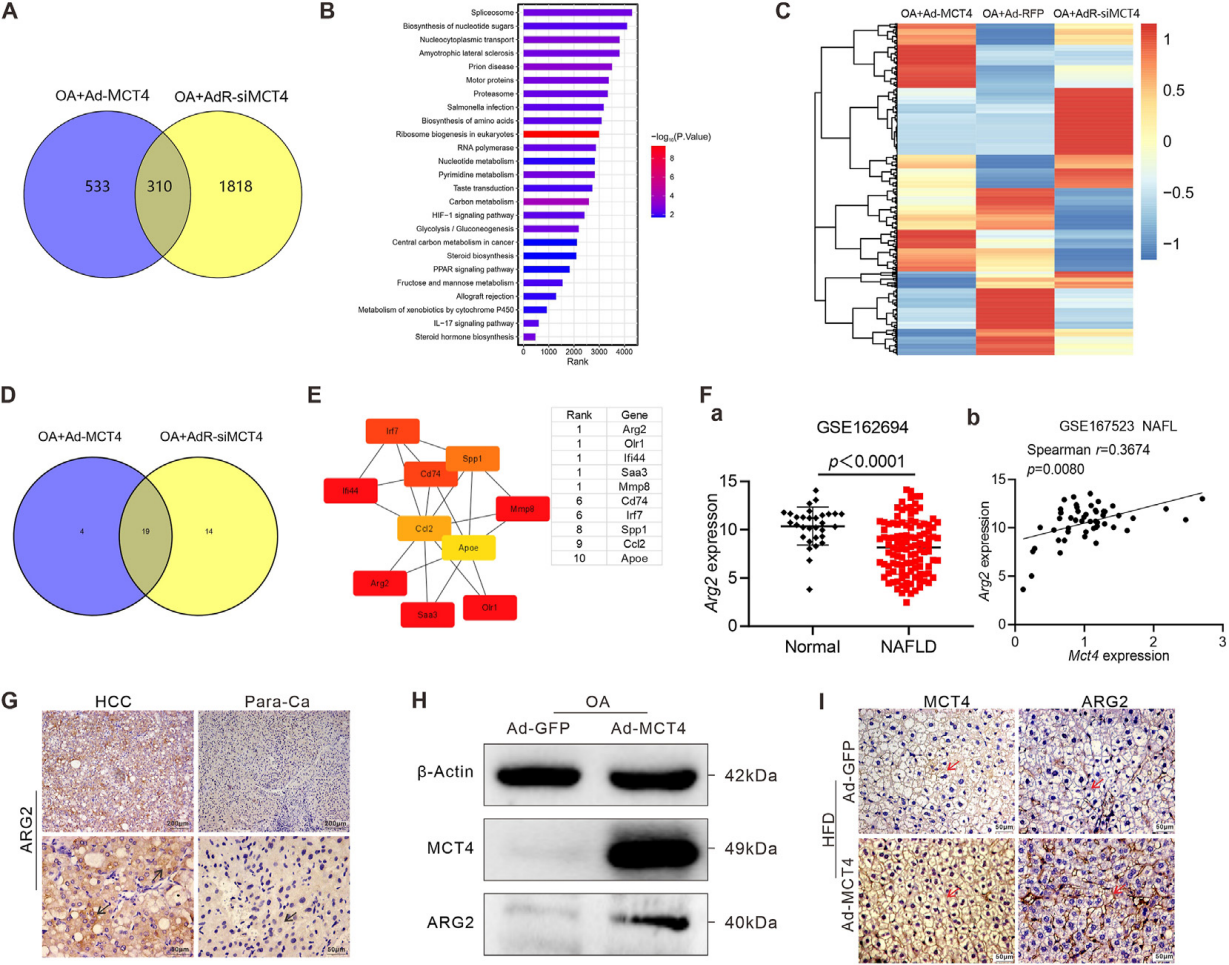
MCT4 regulates hub genes interactions to influence hepatic lipid metabolism. Subconfluent iHPx cells were infected with Ad-MCT4, AdR-siMCT4, or Ad-RFP, and stimulated with 0.05 mM oleic acid for 48 h. Total RNA was collected for RNA-sequencing analysis. **(A)** Venn Diagram of the differentially expressed genes (DEGs). **(B)** Gene set enrichment analysis (GSEA) of all expressed genes (MCT4/siMCT4). **(C)** Clustering analysis of FPKM values of 556 DEGs. **(D)** Venn diagram of partial DEGs verified by touchdown-quantitative PCR. **(E)** The network landscape generated by Cytoscape indicates a potential interaction relationship (colors indicate the importance of genes). **(F)** Scatter plot for *Arg2* TPM of Normal (*n* = 31) and NAFLD (*n* = 112) in GSE162694 dataset (a). Unpaired, two-sided Mann–Whitney U test *P*-value are depicted in the plot, and the plot shows the medians (black line), standard deviation, and *P*-value. Correlation analysis of *Arg2* level with *Mct4* in human NAFL liver samples (GSE167523, *n* = 51) (b). *r* and *P*-value were obtained via two-tailed nonparametric Spearman's test, and the plot shows the linear regression line (black line) and *r* and *P*-value. **(G)** Immunohistochemical staining of ARG2 expression in liver samples in Figure 1E. Representative positive stains are indicated with black arrows (100 × and 400 × ). **(H)** Subconfluent iHPx cells were infected with Ad-MCT4 or Ad-GFP, and stimulated with oleic acid, and total cell lysate was subjected to western blotting analysis to assess MCT4 and ARG2 expression at 72 h. Each assay condition was done in triplicate, and representative images were shown. **(I)** Immunohistochemical staining of MCT4 and ARG2 expression in liver tissues retrieved in Figure 4. Representative positive stains are indicated with red arrows (400 × ). MCT4, monocarboxylate transporter 4; NAFLD, non-alcoholic fatty liver disease; NAFL, non-alcoholic fatty liver; FPKM, fragments per kilo base per million mapped reads; TPM, transcript per million; ARG2, arginase 2.

To understand the potential mechanisms underlying MCT4 exerting its protective effects in hepatic steatosis, we performed mRNA-sequencing transcriptome analyses in the hepatocytes treated with MCT4 overexpression or siMCT4 silencing. We found 843 DEGs and 2128 DEGs in the Ad-MCT4 and AdR-siMCT4 groups, respectively. The Venn diagram analysis indicated that 310 DEGs were shared by two groups (Fig. 5A). Further KEGG pathway analysis of these 310 DEGs revealed that the metabolic pathway was the first to be enriched, especially for the pathways related to NAFLD such as TNF signaling pathway, IL-17 signaling pathway, PI3K-AKT signaling pathway, Wnt signaling pathway, and JAK-STAT signaling pathway, were all enriched (Fig. S4A). Consistently, GSEA enrichment analysis revealed that the HIF-1 signaling pathway, steroid biosynthesis, PPAR signaling pathway, IL-17 signaling pathway, and steroid hormone biosynthesis were enriched (Fig. 5B). Interestingly, many inflammatory response processes associated with NAFLD were identified in GO enrichment analysis on the top 50 biological processes (Fig. S4B), indicating that MCT4 may impact NAFLD progression by regulating both metabolism and inflammation.

Subsequently, we used more stringent thresholds (*i.e.*, *P* < 0.05 and log_2_ |fold change| > 1 in Ad-MCT4 compared with Ad-RFP; *P* < 0.05 and log_2_ |fold change| > 1.5 in AdR-siMCT4 compared with Ad-RFP), and identified 556 DEGs and clustered them (Fig. 5C), and revealed that MCT4 and siMCT4 regulated different transcriptomic profiles. Further clustering of lipolysis, lipogenesis, and regulatory genes showed that lipid transcriptome genes were significantly changed in the Ad-MCT4 group (Fig. S4C). We selected 49 DEGs for TqPCR validation analyses (Table S2) and Venn diagram analysis showed that 19 DEGs were shared between two groups (Table S3 and Fig. 5D), which could be further clustered according to the mRNA expression of the DEGs (Fig. S4D). Specifically, in the Ad-MCT4 group, *Apoe*, *Spp1*, *Mmp8*, and *Arg2* were expressed at high levels, whereas *Lum*, *Olr1*, *Prkcg*, *Fst*, *Abhd3*, *Dgke*, *Irf7*, *Cd74*, and *Steap4* were expressed at lower levels than in the AdR-siMCT4 group (Fig. S4D). Except for *Dgke* and *Abhd3*, those genes were previously identified as altered in the progression of NAFLD.^53^, ^54^, ^55^, ^56^, ^57^, ^58^, ^59^, ^60^, ^61^, ^62^, ^63^, ^64^ The interaction network between the above 19 DEGs encoding proteins was constructed using the STRING database and then visualized by Cytoscape. The cytoHubba-clustering coefficient plugin was also used to identify hub genes; 10 hub genes were obtained, of which *Arg2*, *Olr1*, *Mmp8, Cd74*, *Irf7*, *Spp1*, and *Apoe* were identified (Fig. 5E).

Specifically, we verified that the expression of the top-ranked *Arg2* was down-regulated and moderately positively correlated with *Mct4* expression in non-alcoholic fatty liver in the GEO dataset (Fig. 5F, panels a & b). Its up-regulation induced by MCT4 overexpression was involved in organic acid, carboxylic acid, and immune response processes (Fig. S4E). Similarly, arginase 2 (ARG2) expression was remarkably up-regulated in liver samples derived from patients with hepatocellular carcinoma and NAFLD (Fig. 5G; Fig. S1B, panel b). Using western blotting analysis, we found that MCT4 overexpression significantly enhanced ARG2 expression in oleic acid-treated mouse hepatocytes (Fig. 5H). Additionally, immunohistochemical staining further confirmed that ARG2 expression was elevated in the high-fat diet-fed group treated with Ad-MCT4 (Fig. 5I; Fig. S4F). Collectively, these results strongly suggest that MCT4 may suppress hepatic steatosis and alleviate the development and progression of NAFLD by regulating multiple lipid metabolism and inflammatory response genes.

## Discussion

NAFLD has high levels of lactate accumulation. Recent studies have demonstrated that elevated lactate levels are accompanied by lactylation, which directly promotes gene transcription.^8^^,^^12^^,^^13^ Mitochondrial pyruvate carrier 1 (MPC1) knockdown increased lactates in hepatocytes, lactylating fatty acid synthase and ultimately down-regulating hepatic lipid accumulation,^12^ while histone lactylation induced hexokinase 2 (HK2) expression and promoted hepatic fibrosis.^65^ Although the mechanism where lactate metabolism plays a role in NAFLD is elusive, NAFLD could be improved by inhibiting lactate production and reducing hepatic triglyceride accumulation.^66^^,^^67^ Previous reports have shown that MCT4 is highly expressed where glycolysis is increased and is used to transport intracellular lactate to the extracellular.^14^ However, it is unclear whether elevated MCT4 levels play a critical role in NAFLD development.

Our results indicate that MCT4 is elevated with increasing NAFLD activity scores and fibrosis scores in the livers of NAFLD patients, whereas its expression is decreased in mouse livers. This difference may be species-related and/or disease status-dependent. NAFLD is a complex disease with multifactorial causes, and different genes are expressed in potentially opposite patterns during the initial and progressive stages of NAFLD.^68^ Unfortunately, we only collected clinical late-stage samples. In our study, we constructed the NAFLD model using age-matched male C57BL/6J mice, while the clinical patient samples were not differentiated by sex and age. Inevitably, there is a significant sex difference in susceptibility to NAFLD in C57BL/6J mice.^69^ Even under the influence of minimal sex and age differences, 50% of genes still show opposite regulation in fatty liver disease in humans and mice.^70^ Therefore, our results may not be generalizable for female mice.

Numerous studies have indirectly implied that disorders of lipid metabolism may be associated with altered MCT4 expression.^16^, ^17^, ^18^, ^19^, ^20^ By directly overexpressing, silencing, and inhibiting MCT4 in hepatocytes *in vitro*, our results showed that MCT4 overexpression decreased genes for lipid synthesis and increased genes involved in lipid catabolism, thus decreasing triglyceride accumulation; whereas, silencing and inhibiting MCT4 promoted the accumulation of lipid metabolites in hepatocytes, which contributes to steatosis. These results suggest that lipid synthesis-associated and lipid catabolism-associated genes may be tightly regulated in a coordinated fashion and that MCT4 will affect the expression of both pathways of lipid metabolism simultaneously. To exclude MCT1 interference, we specifically inhibited MCT1 expression using BAY-8002, consistent with previous reports that MCT1 reduction effectively inhibited lipid biosynthesis,^71^^,^^72^ showing the opposite outcomes to that of MCT4. When we constructed a NAFLD model overexpressing MCT4 in mice, the high expression of MCT4 significantly reduced hepatic lipid-regulated genes and intrahepatic deposition of free fatty acids, glucose, and lactates, alleviating hepatocellular steatosis. However, by detecting the levels of metabolites in the serum, we found that these serum indices basically stayed within the normal level, which was not consistent with the results of morphological analyses. This may be because the serum indices may not be sensitive indicators of the liver's pathological state.

Fibrosis is another feature of NAFLD that drives the transition from hepatic steatosis to non-alcoholic steatohepatitis. Inflammation is the driving cause of fibrosis, and although this study did not specifically analyze changes in inflammatory markers, inflammation-related genes were also found after mRNA sequencing, significantly enriched in inflammatory pathways. *Irf7*, *Ccl2*, *Mmp8*, *Ifi44*, and other genes were similarly found to change in our TqPCR validation experiments. Our previous study found that MCT4 inhibition highly expressed interferon-gamma (IFN-γ) and α-smooth muscle actin (a-SMA) at both the gene and protein levels, promoting liver fibrosis.^21^ Similarly, a study found elevated levels of MCT4 during anti-hepatic fibrosis with the energy blocker 3-bromopyruvate.^73^ Meanwhile, MCT4 may exert cardioprotective effects by regulating oxidative stress and inflammatory responses.^74^^,^^75^ Our results indicate that MCT4 may play a protective role in NAFLD progression.

To further understand the potential mechanisms by which MCT4 alleviates NAFLD, we performed mRNA sequencing and analyzed the transcriptomic landscape of hepatocytes after overexpression or silence of MCT4. Our result showed that MCT4 regulated NAFLD-related metabolic pathways, including PPAR, HIF-1, TNF, IL-17, PI3K-AKT, Wnt, and JAK-STAT signaling pathways, as well as enriched multiple inflammation-related biological processes. In our validation experiments, overexpression and silence of MCT4 regulated lipid metabolism through overlapping but distinct DEGs, respectively, and significantly modulated genes affecting metabolism and inflammatory responses. Not surprisingly, the Ad-MCT4 group expressed high levels of the genes that alleviate fatty liver including *Spp1*,^53^ *Apoe*,^54^ and *Arg2*,^53^^,^^55^ whereas the genes that promote fatty liver including *Lum*,^56^ *Olr1*,^64^^,^^76^ *Prkcg*,^57^ *Fst*,^61^ *Irf7*,^58^ and *Cd74*^59^^,^^62^ were less expressed than those in AdR-siMCT4 group. Interestingly, *Mmp8* is highly expressed in the Ad-MCT4 group and has been reported to influence cholesterol efflux and to be highly expressed in NAFLD,^60^^,^^77^ whereas its role in NAFLD has not been clearly investigated, suggesting that the inflammation–metabolism interface displays a complex network of regulatory mechanisms. Among them, *Arg2*, *Olr1*, *Mmp8*, *Cd74*, *Irf7*, *Spp1*, and *Apoe* were identified as hub genes, and *Arg2* was considered the most critical regulatory gene. Hepatocyte ARG2 was found to prevent hepatic and peripheral lipid accumulation and hepatic inflammatory response and reduce liver injury.^78^^,^^79^ Furthermore, *Arg2* is a downstream gene of interleukin-10, and interleukin-10-mediated up-regulation of ARG2 exerts anti-inflammatory effects.^80^ Subsequently, we found that MCT4 expression was positively correlated with ARG2 at both gene and protein levels, which is consistent with ARG2 playing a protective role in NAFLD. It is worth mentioning that secreted phosphoprotein 1 (SPP1) overexpression was recently found to up-regulate ARG2-induced fatty acid oxidation in hepatocytes, which has a protective effect on hepatocytes,^53^ both genes were highly expressed after MCT4 overexpression in this study. Thus, our results suggest that MCT4 may affect NAFLD progression by driving multiple genes interactions that regulate hepatic lipid metabolism and inflammatory responses.

In summary, we found that MCT4 played an important role in NAFLD progression. Both *in vivo* and *in vitro* experiments demonstrated that MCT4 up-regulation inhibited lipid accumulation in hepatocytes by down-regulating a series of hepatic lipid-regulated genes, whereas down-regulation of MCT4 expression promoted hepatic lipid accumulation. Mechanistically, MCT4 reduced steatosis by regulating anabolic and catabolic genes and inflammatory pathways, thereby reducing NAFLD progression. Nevertheless, there are limitations to our current study. NAFLD was not subdivided when analyzing the clinical samples as it is a progressive disease and there is a lack of sources of clinical early-stage samples; we will subsequently collect clinical samples over time for further analysis. Nonetheless, our study provides an important preliminary exploration of the molecular mechanisms whereby MCT4 functions in NAFLD for further investigation.

## Conclusions

To explore the relationship between MCT4 and NAFLD, we analyzed the differences in lipid accumulation *in vivo* and *in vitro* by overexpressing and silencing MCT4 expression directly in hepatocytes, demonstrating that MCT4 could regulate both lipid anabolism and catabolism. The high expression of MCT4 significantly down-regulated the hepatic lipid-related genes and the intrahepatic triglyceride/free fatty acid deposition and mitigated hepatocytic steatosis. Transcriptomic analysis showed that MCT4 regulated PPAR, HIF-1, TNF, IL-17, PI3K-AKT, Wnt, and JAK-STAT signaling pathways, as well as multiple inflammation-related biological processes by regulating multiple genes interactions such as *Arg2*, *Mmp8*, *Spp1*, *Apoe*, *Olr1*, *Cd74*, and *Irf7*. Our results revealed that exogenous MCT4 may reduce liver steatosis and ultimately attenuate NAFLD. Collectively, our findings delineate the potential functions and mechanisms of MCT4 in NAFLD and provide novel insights into the clinical perspectives of NAFLD treatment.

## CRediT authorship contribution statement

**Yannian Gou:** Writing – review & editing, Writing – original draft, Visualization, Validation, Methodology, Formal analysis, Data curation, Conceptualization. **Aohua Li:** Writing – review & editing, Writing – original draft, Project administration, Formal analysis, Data curation. **Xiangyu Dong:** Writing – review & editing, Methodology, Formal analysis. **Ailing Hao:** Writing – review & editing, Methodology, Data curation. **Jiajia Li:** Writing – review & editing, Resources, Methodology, Formal analysis. **Han Xiang:** Writing – review & editing, Resources, Formal analysis. **Saidur Rahaman:** Writing – review & editing, Resources, Methodology. **Tong-Chuan He:** Writing – review & editing, Writing – original draft, Validation, Supervision, Resources, Project administration, Methodology, Funding acquisition, Formal analysis, Data curation, Conceptualization. **Jiaming Fan:** Writing – review & editing, Writing – original draft, Validation, Supervision, Resources, Project administration, Methodology, Funding acquisition, Formal analysis, Data curation, Conceptualization.

## Ethics declaration

The use and care of experimental animals were approved by the Research Ethics and Regulations Committee of Chongqing Medical University, Chongqing, China. All experimental procedures followed the approved guidelines.

## Funding

The reported study was supported in part by research grants from the 10.13039/501100001809Natural Science Foundation of China (No. 82102696 to J.F.), the Chongqing Natural Science Foundation of China (No. 2024NSCQ-MSX0073 to J.F.), and the 10.13039/100000002US National Institutes of Health (No. CA226303 to T.C.H.). This project was also supported in part by The University of Chicago Cancer Center Support Grant (No. P30CA014599) and the National Center for Advancing Translational Sciences of the US National Institutes of Health (No. UL1 TR000430). T.C.H. was supported by the Mabel Green Myers Research Endowment Fund and The University of Chicago Orthopaedics Alumni Fund. Funding sources were not involved in the study design; in the collection, analysis, and interpretation of data; in the writing of the report; and in the decision to submit the paper for publication.

## Conflict of interests

Tong-Chuan He is the member of *Genes & Diseases* Editorial Board. To minimize bias, he was excluded from all editorial decision-making related to the acceptance of this article for publication. The remaining authors declare no conflict of interests.

## Appendix A Supplementary data

Multimedia component 1 Multimedia component 1

Table S1 List of touchdown-quantitative PCR primers. Table S1

Table S2 Fragments per kilo base per million mapped reads of differentially expressed genes. Table S2

Table S3 Touchdown-quantitative PCR validation of differentially expressed genes. Table S3
