# Supplementary material for: Lactate transporter MCT4 regulates the hub genes for lipid metabolism and inflammation to attenuate intracellular lipid accumulation in non-alcoholic fatty liver disease
Source: Genes Dis. 2025 Feb 15;12(4):101554. doi: 10.1016/j.gendis.2025.101554 (PMC12052676; doi:10.1016/j.gendis.2025.101554)
Supplement: Multimedia component 1 [file mmc1.docx]

**Lactate transporter MCT4 regulates the hub genes for lipid metabolism and inflammation to attenuate intracellular lipid accumulation in non-alcoholic fatty liver disease (NAFLD)**

**Running Title: Lactate transporter MCT4 alleviates hepatic steatosis**

Yannian Gou^a,b,1^, Aohua Li^a,1^, Xiangyu Dong^a^, Ailing Hao^a^, Jiajia Li^a^, Han Xiang^a^, Saidur Rahaman^a^, Tong-Chuan He^c,^* and Jiaming Fan^a,b,^*

1. Ministry of Education Key Laboratory of Diagnostic Medicine, and Department of Clinical Biochemistry, School of Clinical Laboratory Medicine, Chongqing Medical University, Chongqing 400016, China
2. Western Institute of Digital-Intelligent Medicine, Chongqing 401329, China
3. Molecular Oncology Laboratory, Department of Orthopaedic Surgery and Rehabilitation Medicine, The University of Chicago Medical Center, Chicago, IL 60637, USA

* Corresponding authors.

^1^ These authors contributed equally to the work.

**Correspondences**

Jiaming Fan, MD, PhD

Ministry of Education Key Laboratory of Diagnostic Medicine

Department of Clinical Biochemistry

School of Clinical Laboratory Medicine

Chongqing Medical University

Chongqing, 400016, China

Tel. 011-86-23-6848 5240

Email: [fanjiaming1988@cqmu.edu.cn](mailto:fanjiaming1988@cqmu.edu.cn)

T.-C. He, MD, PhD

Molecular Oncology Laboratory

The University of Chicago Medical Center

Chicago, IL 60637, USA

Tel. (773) 702-7169

Fax: (773) 834-4598

Email: [tche@uchicago.edu](mailto:tche@uchicago.edu)

**Supplementary Materials**


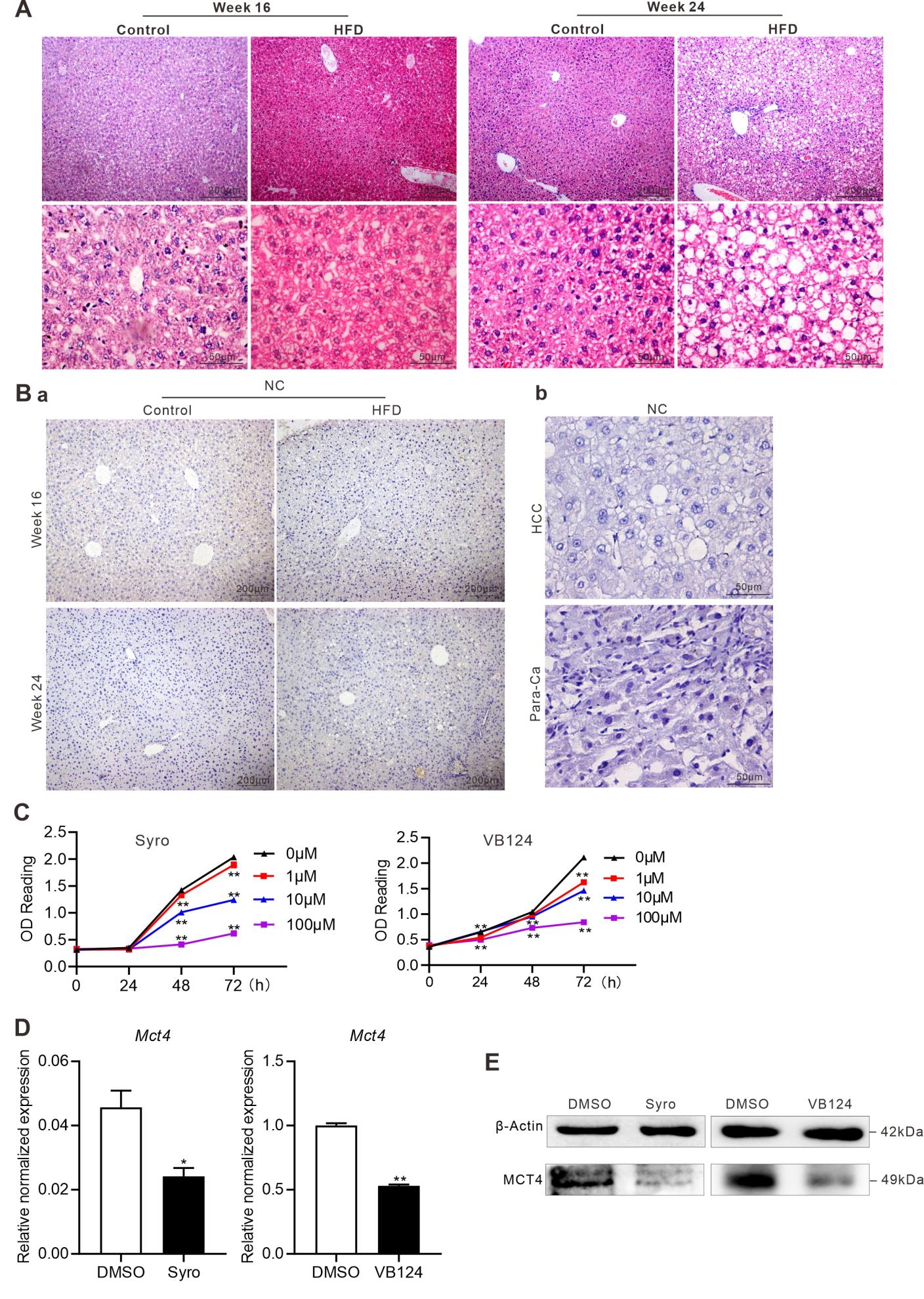


**Figure S1** **Hematoxylin & eosin staining, immunohistochemical negative controls, and the optimal inhibitory capacity of Syrosingopine and VB124 in iHPx cells.** **(A)** Paraffin sections of liver samples in Figure 1D were subjected to hematoxylin & eosin staining (100× and 400×). **(B)** Immunohistochemical negative controls for Figure 1D, E. The paraffin sections of non-alcoholic fatty liver disease (NAFLD) mouse (a) and hepatocellular carcinoma (HCC) patients (b) liver samples were subjected to immunohistochemical staining without any primary antibody (100× or 400×). **(C)** The optimal inhibitory concentration of Syrosingopine and VB124 in iHPx cells. iHPx cells were seeded in 96-well cell plates and treated with 0 μM, 1 μM, 10 μM, and 100 μM Syrosingopine or VB124, followed by WST-1 assay at 0 h, 24 h, 48 h, and 72 h. ***P* < 0.01, 0 μM inhibitor treatment group versus 1 μM, 10 μM, or 100 μM inhibitor treatment group at the indicated time points. **(D)** The inhibitory effect of Syrosingopine and VB124 in iHPx cells. Subconfluent iHPx cells were infected with 10 μM Syrosingopine, 10 μM VB124, or DMSO for 36 h. Total RNA was isolated and subjected to touchdown-quantitative PCR analysis of *Mct4* expression. **P* < 0.05, ***P* < 0.01, inhibitors versus DMSO. **(E)** Western blotting analysis was used to assess the inhibitory effect of Syrosingopine and VB124 in iHPx cells at 72 h. Each assay condition was done in triplicate, and representative images were shown. MCT4, monocarboxylate transporter 4.


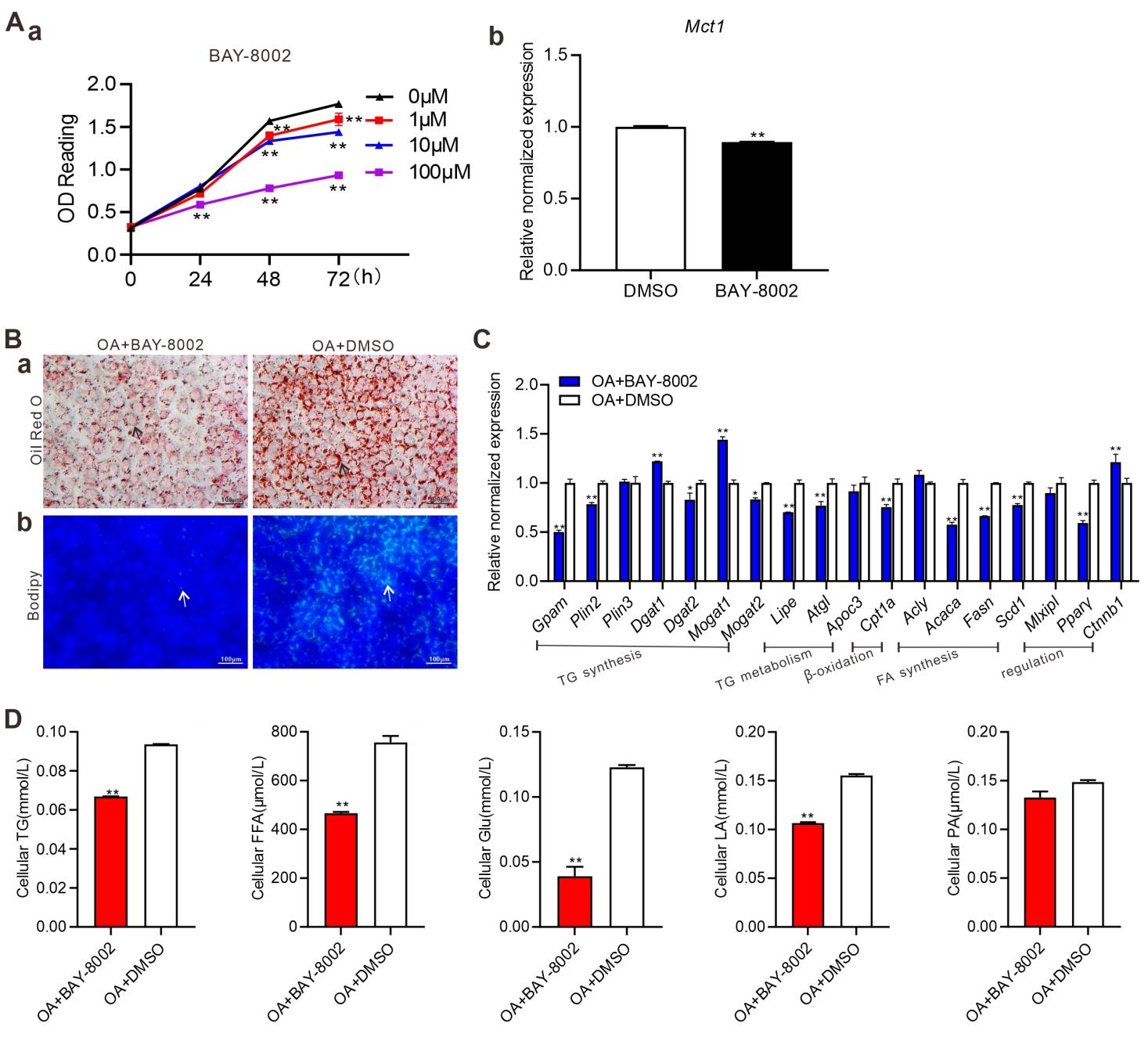


**Figure S2 BAY-8002 decreases lipid accumulation in hepatocytes. (A)** The inhibitory effect of BAY-8002 in iHPx cells. iHPx cells were seeded in 96-well cell plates and treated with 0 μM, 1 μM, 10 μM, and 100 μM BAY-8002, followed by WST-1 assay at 0 h, 24 h, 48 h, and 72 h (a). ***P* < 0.01, 0 μM BAY-8002 treatment group versus 1 μM, 10 μM, or 100 μM BAY-8002 treatment group at the indicated time points. Subconfluent iHPx cells were infected with 10 μM BAY-8002 or DMSO for 36 h. Total RNA was isolated and subjected to touchdown-quantitative PCR analysis of *Mct1* expression (b). ***P* < 0.01, BAY-8002 versus DMSO. **(B)** Subconfluent iHPx were treated with 10 µM BAY-8002 (DMSO as the solvent control) and oleic acid (OA) for 5 days, and subjected to oil red O staining (a) and bodipy 493/503 staining (b). Representative lipid droplets were indicated with arrows (200×). **(C)** Touchdown-quantitative PCR analysis of the expression of the genes involved in lipid anabolism and catabolism, and transcriptional regulation in hepatocytes after 36 h of BAY-8002 and OA treatment. Expression of each target gene was calculated as a relative expression to *Gapdh*. **P* < 0.05, ***P* < 0.01, OA + BAY-8002 versus OA + DMSO. **(D)** The cellular triglyceride (TG), free fatty acid (FFA), glucose (Glu), lactate (LA), and pyruvate (PA) levels were measured on day 5 of BAY-8002 and OA treatment. ***P* < 0.01, OA + BAY-8002 versus OA + DMSO. Each assay condition was done in triplicate, and representative images were shown.


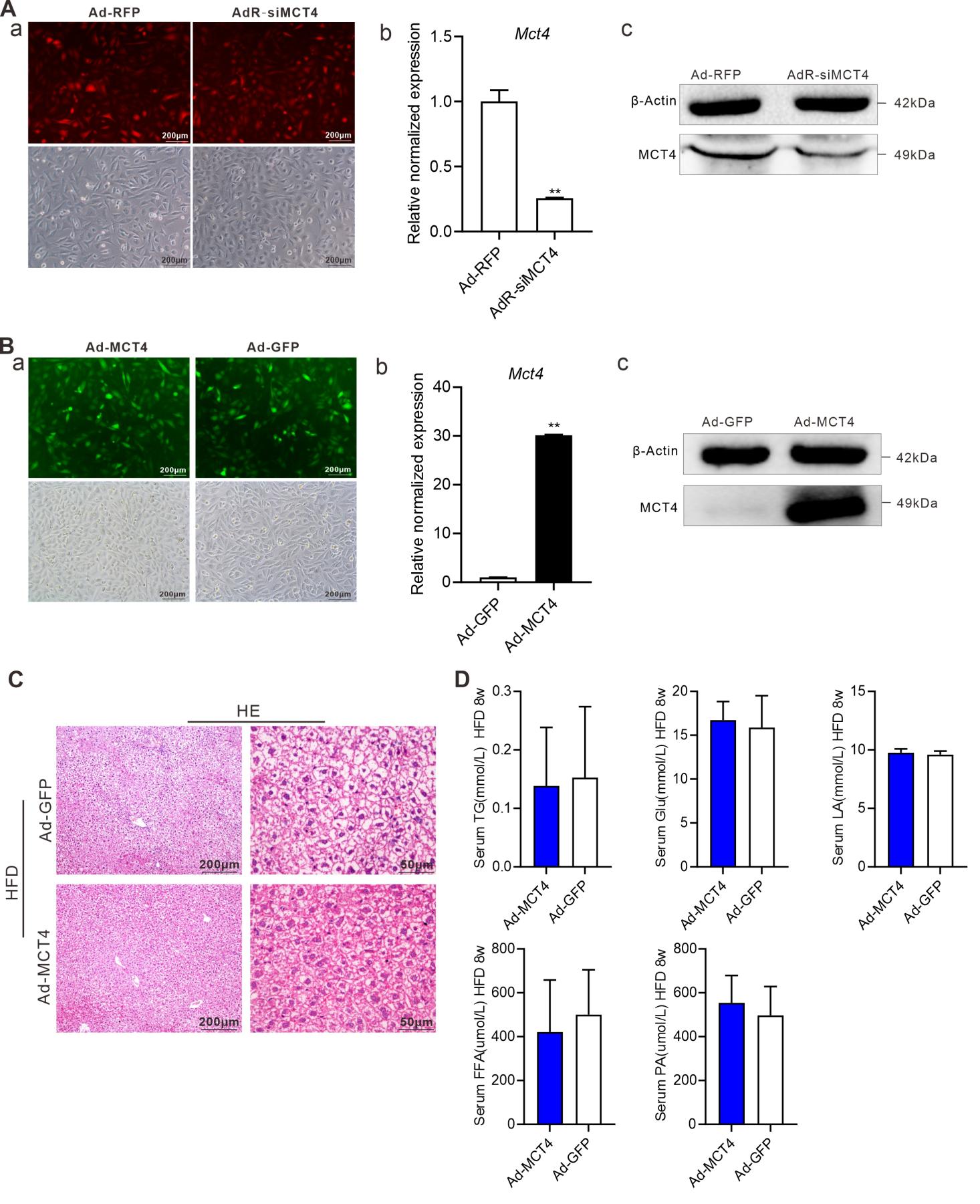


**Figure S3** **Overexpression and silencing of MCT4 in iHPx cells and morphologic and serologic analyses of nonalcoholic fatty liver disease mouse model.** **(A)** Silencing of MCT4 in iHPx cells. Subconfluent iHPx cells were infected with Ad-RFP or AdR-siMCT4 for 48 h. The fluorescence signal was recorded (100×) (a). Total RNA was isolated for touchdown-quantitative PCR analysis of the expression of *Mct4* (b). ***P* < 0.01, AdR-siMCT4 group versus Ad-RFP group. Alternatively, western blotting was used to detect changes in MCT4 protein levels at 72 h (c). **(B)** Overexpression of MCT4 in iHPx cells. Subconfluent iHPx cells were infected with Ad-GFP or Ad-MCT4 for 48 h. The fluorescence signal was recorded (100×) (a). Total RNA was isolated for touchdown-quantitative PCR analysis of the expression of *Mct4*. ***P* < 0.01, Ad-MCT4 group versus Ad-GFP group. Alternatively, western blotting was used to detect changes in MCT4 protein levels at 72 h (c). **(C)** Paraffin sections of liver samples in Figure 4 were subjected to hematoxylin & eosin staining (100× and 400×). **(D)** The serum triglyceride (TG), free fatty acid (FFA), glucose (Glu), lactate (LA), and pyruvate (PA) levels in Figure 4 were measured. MCT4, monocarboxylate transporter 4.


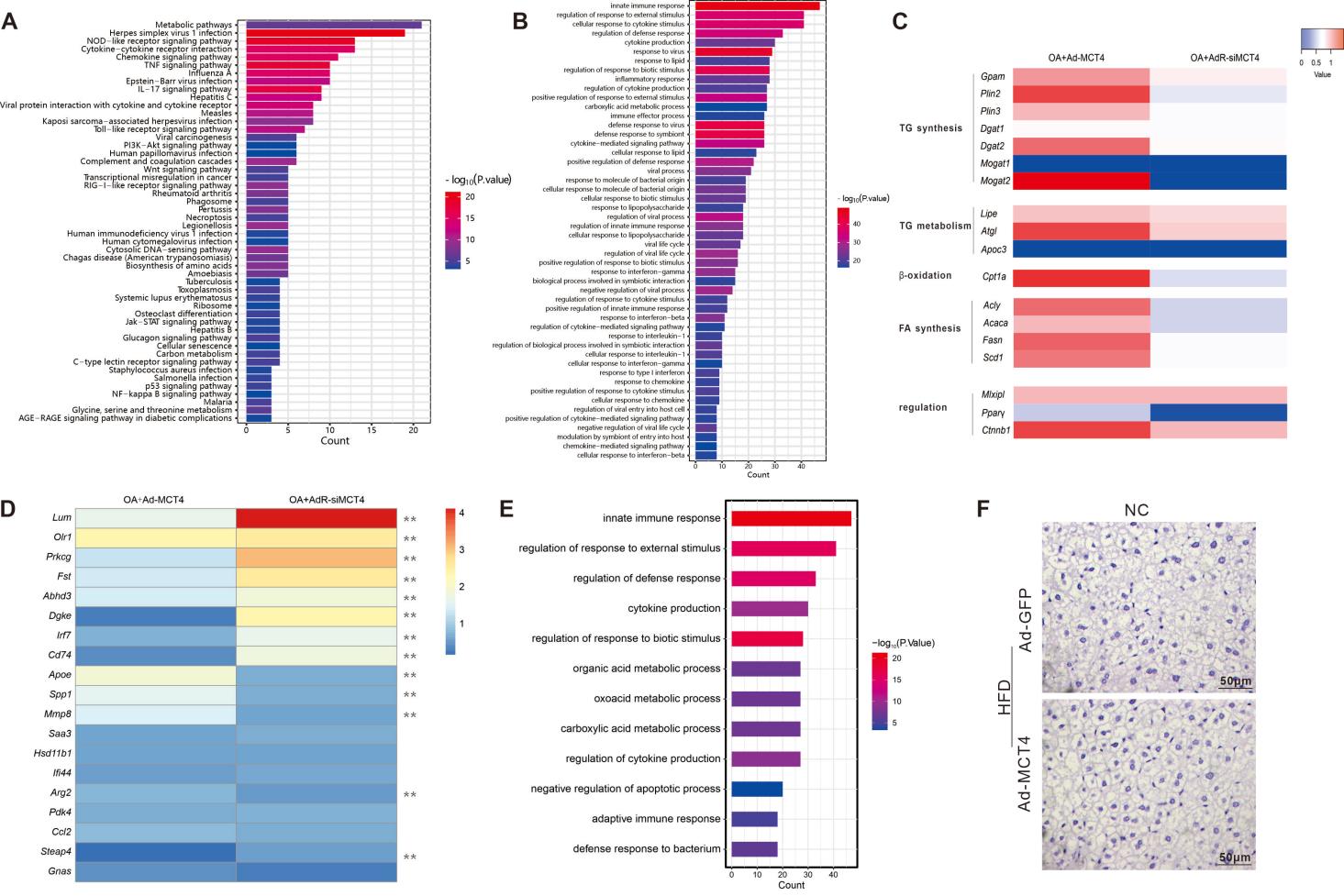


**Figure S4** **The transcriptomic landscape of the iHPx cells stimulated by MCT4 overexpression and silencing.** **(A)** Kyoto Encyclopedia of Genes and Genomes (KEGG) enrichment of the top 50 signaling pathways for the 310 differentially expressed genes in Figure 5A. **(B)** Gene Ontology (GO) enrichment of the top 50 biological processes for the 310 differentially expressed genes in Figure 5A. **(C)** FPKM cluster analysis of genes involved in lipid anabolism and catabolism. **(D)** Heatmap visualization of the expression of consensus 19 differentially expressed genes in Figure 5D. ***P* < 0.01, OA + Ad-MCT4 group versus OA + AdR-siMCT4 group. **(E)** The GO biological processes involving *Arg2* in Figure S4B. **(F)** Paraffin sections of liver samples in Figure 5I were subjected to immunohistochemical staining without any primary antibody (400×). MCT4, monocarboxylate transporter 4; FPKM, fragments per kilo base per million mapped reads; *Arg2*, arginase 2.
